# The Need for Head Space: Brachycephaly and Cerebrospinal Fluid Disorders

**DOI:** 10.3390/life11020139

**Published:** 2021-02-12

**Authors:** Clare Rusbridge, Penny Knowler

**Affiliations:** 1 Faculty of Health & Medical Sciences, School of Veterinary Medicine, University of Surrey, Guildford GU2 7AL, UK; s.knowler@surrey.ac.uk; 2 Fitzpatrick Referrals, Godalming GU7 2QQ, UK

**Keywords:** ventriculomegaly, hydrocephalus, Chiari malformation, syringomyelia, canine, craniosynostosis, supracollicular fluid collection, quadrigeminal cistern, lateral aperture, sleep disordered breathing, brachycephalic obstructive airway disease

## Abstract

Brachycephalic dogs remain popular, despite the knowledge that this head conformation is associated with health problems, including airway compromise, ocular disorders, neurological disease, and other co-morbidities. There is increasing evidence that brachycephaly disrupts cerebrospinal fluid movement and absorption, predisposing ventriculomegaly, hydrocephalus, quadrigeminal cistern expansion, Chiari-like malformation, and syringomyelia. In this review, we focus on cerebrospinal fluid physiology and how this is impacted by brachycephaly, airorhynchy, and associated craniosynostosis.

## 1. Introduction

Brachycephalic dogs and cats have proved to be increasingly popular since they were introduced to Europe in Victorian times. This rising popularity defies a high prevalence of conformation-related morbidity, including breathing, ocular, and neurological disorders [1,2]. Brachycephaly in domestic pets is a consequence of selecting for juvenile characteristics of a flattened face and a rounded head [3]. Calvarial doming associated with wide zygomatic arches and a wide, flattened, or convex palate is compensation for premature closure of skull-base sutures, including the basispheno-presphenoid synchondrosis and spheno-occipital synchondrosis [4,5]. Brachycephalic dogs and cats also have premature closure of other cranial and facial sutures when compared to other skull types [4,6]. The skull functions to house and protect the brain and five special sense organs (vision, smell, taste, hearing, and balance). It also facilitates breathing and eating. Extreme shortening of the cranium (brachycephaly) with shortened and a dorsally rotated rostrum (airorhynchy) impact these vital functions. Brachycephalic dogs and cats are predisposed to cerebrospinal fluid (CSF) circulation disorders, such as ventriculomegaly, hydrocephalus, quadrigeminal cistern expansion, Chiari-like malformation, and syringomyelia. Disorders of CSF and hydrocephalus are among the earliest recognized neurological conditions in dogs [7], and the predisposition of toy breed brachycephalic dogs to ventriculomegaly and hydrocephalus is evident from the time that they first became fashionable, as evidenced by skulls in museum collections [8]. This article reviews the possible mechanisms behind that predisposition.

## 2. Effect of Brachycephaly on Brain Conformation, Compliance, and Pulsatility

The skull develops in response to the expanding brain mass. The ultimate shape of the skull is determined by the growth of the cranial sutures. The sutures are fibrous joints between calvarial bones, which develop at the site of dural reflections, where the inner dural layer descends into the brain, forming septa, which divide the brain into compartments (for example, the falx cerebri and the tentorium cerebelli) [9,10]. Suture growth is regulated by morphogenetic peptide growth factors and chemokines secreted from the dura. The dura has a mechanosensory capacity, and it responds to mechanical stress from the expanding brain and, in so doing, controls skull growth [9,10]. If one or more sutures close prematurely (craniosynostosis), then compensatory growth occurs in a plane parallel to the fused suture with minimal growth in a perpendicular plane [11]. However, in more extreme brachycephaly and compound craniosynostosis, the ability of the skull to compensate may be overcome, resulting in neuroparenchymal overcrowding. Brain conformation mirrors the cranial cavity, reflecting not only rostrocaudal shortening, but rotation on the medial lateral axis with ventral displacement and reduction of the olfactory bulbs [12,13]. The overcrowding of neuroparenchymal tissue and loss of the buffering subarachnoid space and cisterns results in reduced compliance of the intracranial compartment. Small increases in volume will result in large increases in intracranial pressure. More important for development of ventriculomegaly is reduced brain compliance and consequently reduce brain pulsatility [14]. Neuroparenchymal compliance and pulsatility are further altered by the developing ventriculomegaly. This alteration in pulsatility may reflect the location of a waveform, as well as timing of propagation and morphology, for example, the hyperdynamic flow and increased pulsatility within the ventricles relative to the subarachnoid space [14].

## 3. Cerebrospinal Fluid Absorption and Movement

Recently, understanding of CSF physiology has shifted from a choroid, plexus, arachnoid, and granulation-centric model of production and absorption to an interstitial, fluid, lymphatic, and vessel-centric model (also referred to as a glymphatic model). In cats and dogs, approximately 40% of CSF is secreted by the choroid plexus into the ventricular spaces; the remainder originates from the brain and spinal interstitial fluid, leptomeningeal and parenchymal capillaries, and ependyma [15,16]. The fluid of the CSF and the brain interstitial space is in continuous flux. From the ventricles, CSF diffuses through the ependyma. In the subarachnoid space, CSF drains into perivascular (Virchow–Robin) spaces, from where it may exchange with the interstitial fluid before emptying into the lymphatic system [17,18,19]. The exchange with interstitial fluid is via and controlled by aquaporins, water transporting pores in the foot processes of astrocytes wrapped around capillaries [20,21,22]. This glymphatic movement of fluids facilitates elimination of waste protein, including β amyloid, hydroxylated cholesterol, and other metabolites. It also facilitates the distribution of neurotransmitters, amino acids, glucose, and lipids [23,24]. Additional functions of the CSF include acting as a hydraulic cushion, buffering changes in intracranial pressure, thereby reducing risk of volume shifts or herniation, affecting brain development by the effect of CSF pressure, regulating neural stem cells by choroid plexus secreted proteins within the CSF, and acting as a port of entry of immune cells into the CNS via the choroid plexus [25]. There is overwhelming evidence that the bulk of CSF is absorbed though perineurial lymphatics (olfactory bulbs and cranial and spinal nerves) and meningeal lymphatic vessels of the skull and sacral spine [18,26]. Previously it was considered that the main site for CSF absorption was through the arachnoid granulations, however, this is insignificant at normal CSF pressure, and more likely serves as an overflow valve mechanism when there is elevated CSF pressure [20]. Although this glymphatic theory of CSF absorption is considered new, it is supported by nineteenth century experiments in dogs. Gustav Schwalbe injected Berlin blue dye into the canine subarachnoid space and showed that the CSF drained though the lymphatics [27].

## 4. Effect of Brachycephaly on the Absorption of CSF through Lymphatics

As the majority of CSF is absorbed through lymphatics in the skull base and via the olfactory bulbs, the balance between CSF production and absorption may be impacted by craniofacial hypoplasia and cranial base shortening.

### 4.1. Reduction of the Cribriform Plate and Nasal Mucosa

There is direct continuity between the subarachnoid space and the perineurial spaces of the olfactory nerve fibers penetrating the cribriform plate. These nerve fibers are encircled by lymphatics, providing one of the main drainage routes of CSF [28,29]. CSF absorption may be compromised if the lymphatic vessel surface area is reduced by brachycephaly and airorhynchy, with the loss of the muzzle and its nasal turbinates and mucosa [30,31]. In a sheep model (created by external ethmoidectomy, removal of the olfactory nerves and mucosa, and sealing the cribriform bone surface with tissue glue, in addition to restricting the craniocervical CSF channels by ligature around the thecal sac at C1/C2) there was elevation in intracranial pressure, elevation in pulse pressure amplitude (and thereby a reduction in neuroparenchymal compliance and altering brain pulsatility), and impairment of intracranial pressure accommodation. In comparison, the sham surgery group (C1/C2 ligature and exposure only) did not have a significant change in intracranial pressure or pulse pressure amplitude [32,33]. In this experimental sheep model, it was necessary to block the cranial cervical CSF channels in addition to obstructing CSF absorption through the olfactory route, as 25% of global CSF transport occurs in the spinal subarachnoid compartment. This can be compared to syringomyelia associated with Chiari-like malformation in the dog, where a combination of craniofacial hypoplasia and craniospinal junction abnormalities predisposes spinal cord fluid cavitation [34].

### 4.2. Reduction of Skull Base Foramen Volume

In addition to olfactory perineural drainage, basal meningeal lymphatics and cranial nerve perineural lymphatics are important for CSF drainage [18,35,36,37,38]. Reduction in the rostral cranial cavity and skull base could reduce the surface area of this lymphatic network. As these lymphatic vessels pass through the skull base foramen, reduction in the skull base could also result in foraminal stenosis and impede drainage. In this regard, one of the most important skull base foramen is the jugular, situated between the temporal and occipital bone. The jugular foramen provides passage to cranial nerves IX, X, XI, sigmoid sinus, and lymphatics. Cavalier King Charles spaniels with syringomyelia associated with Chiari-like malformation have smaller volume jugular foramina compared to Cavalier King Charles spaniels without syringomyelia [39]. However, direct causality between smaller jugular foramen and syringomyelia has not been proven.

## 5. Cranial Venous Stenosis

The skull base foramina allow passage of the cranial venous sinuses. Reduction of the skull base and stenosis could therefore impede venous drainage and increase intracranial venous pressure, reducing venous resorption of cerebrospinal fluid. For example, stenosis of the jugular foramen will impede drainage through the sigmoid sinus and therefore the transverse sinus [40,41].

However, craniosynostosis may be associated with a primary venous abnormality. Cavalier King Charles spaniels with syringomyelia associated with Chiari malformation have reduced volume caudal cranial fossa dorsal sinuses [42]. Mouse models and humans affected by craniosynostosis may have stenosis of the cerebral veins independent of the skull malformation. These persist after skull expansion surgery, and may lead to elevated intracranial pressure. It has been shown that the bone morphogenic protein (BMP) signaling pathways from skull preosteoblasts and periosteal dura influence vascular development, and it is hypothesized that genetic mutations that affect BMP pathways may influence cerebral vein development and physiology [43,44].

## 6. Effect of Brachycephaly on Respiration and Sleep

Brachycephalic dogs with short muzzles are predisposed to the conformation-related respiratory disorder brachycephalic obstructive airway syndrome (BOAS) [45]. Soft tissue and skull disproportion result in the narrowing or obstruction of the airway through the nose and pharynx. The resulting increased airway resistance and long-term negative pressure gradients lead to secondary changes, such as eversion of the laryngeal saccules and the collapse of the larynx [46] and pharynx [47]. Brachycephalic dogs with BOAS have clinical biomarkers of chronic intermittent hypoxia, like humans affected by sleep apnea syndrome [48]. Like humans, acquired conformational factors, such as obesity and increased neck girth, increase the risk of breathing disorders [49].

Respiration drives CSF movement through the ventricular system [50]. The systolic pulse contributes to a lesser degree [51,52]; the pulse of choroid plexus secretion is not relevant [16]. There is a bidirectional flow with the rostral movement of CSF during deep inhalation and caudal movement during deep exhalation [50,53]. Apnea impedes CSF movement, and apnea-induced negative intrathoracic pressure will reduce CSF drainage through the venous sinuses [54,55]. This has obvious implications for brachycephalic dogs with BOAS, especially if they have sleep-disordered breathing. Intracranial pressure increases during sleep, and disordered sleep will also affect glymphatic drainage, which is more active during sleep [21,55,56].

### 6.1. Upper Respiratory Tract Conformation

The oropharynx, larynx, and hyoid are suspended from the cranial base. Skull base shortening may displace these tissues and the tongue, caudally affecting swallowing and respiration, especially if there are other conformation problems reducing airway space. In rats and humans, these conformational changes are associated with obstructive sleep apnea and sleep-disordered breathing [57,58].

### 6.2. Negative Intrathoracic Pressure

Animals with severe BOAS have increased thoracic effort with increased inflation of the lungs and use of accessory abdominal musculature [46]. This decreased intrathoracic pressure will reduce venous return to the heart, predispose intracranial venous hypertension, and reduce CSF drainage [55]. Long-term negative pressure gradients may also influence the development of syringomyelia and the velocity of fluid moving within the syrinx.

## 7. Effect of Brachycephaly on Craniospinal Junction Conformation

**Figure 1:**
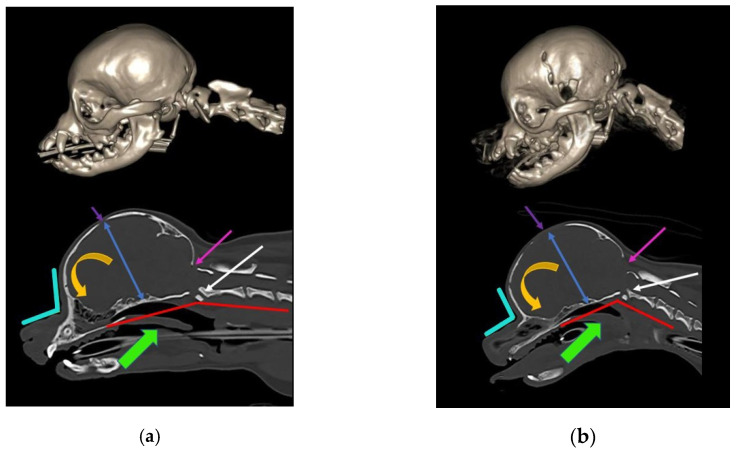
Skull and craniocervical junction changes with extreme brachycephaly. Reconstructed and midsagittal CT of two female sibling Chihuahuas aged one year old. (**a**) Less extreme 2.5 kg female Chihuahua, sibling to (**b**). (**b**) More extreme miniaturized and brachycephalic 1.6 kg female Chihuahua, sibling to (**a**). With increasing brachycephaly, the angle of stop (junction between frontal and nasal bones; aqua angle) becomes more acute. With more extreme brachycephaly, ventral brain rotation is more pronounced (orange arrow). There is a relative increase in the cranium height (blue arrow), and the molera (persistent bregmatic fontanelle; purple arrow) is wider. Altered occipital bone conformation changes the angle between the skull base and the cervical vertebrae, resulting in a cervical flexure (red line) and dorsal tipping of the odontoid peg into the spinal cord (white arrow). The oropharynx is displaced caudally (green arrow). There is also a Chiari-like malformation with a small caudal fossa, reduced occipital crest, and a short, more vertical supraoccipital bone (pink arrow). The supraoccipital bone has failed to ossify ventrally. The atlas is closer to the skull, contributing to the craniocervical junction overcrowding (images created by C. Rusbridge and S.P. Knowler).

The skull base forms the lower part of the foramen magnum, which accommodates the craniospinal junction. Premature closure of the spheno-occipital synchondrosis alters the angulation between the skull base and the cervical vertebrae [59,60,61]. The skull base is flexed dorso-caudally, which is mirrored by a corresponding “concertina” brain flexure (so-called sphenoid flexure) [61]. The occipital condyles and the foramen magnum rotate rostrally and ventrally [60]. This craniocervical junction angulation conformation results in ventral deviation of the neck (so-called cervical flexure), with odontoid peg angulation and kinking of the craniospinal junction (Figure 1). This is comparable to basilar invagination in humans. In dogs, this conformation change, in addition to brachycephaly, predisposes syringomyelia secondary to Chiari-like malformation [61,62,63]. Malformation and malalignment of the craniocervical junction can result in obstruction of vascular and CSF pathways [64]. CSF moves caudally from the cranium into the spinal compartment, and is absorbed, together with CSF produced by the spinal cord, by lymphatics in the sacral spine and along nerve root sleeves in the cervical region [26,52]. CSF also moves rostrally from the spinal into the cranial compartment during deep inhalation [50,53] and diastole [52]. It is proposed that dissociation of CSF flow in the cranial and spinal compartments predisposes syringomyelia [65,66].

## 8. Effect of Brachycephaly on Conformation of the Lateral Aperture of the Fourth Ventricle

**Figure 2:**
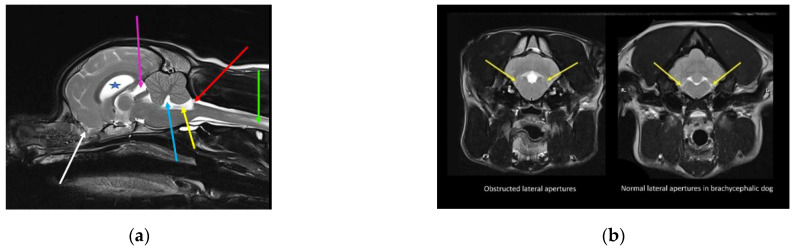
Possible lateral aperture obstruction in a three-year-old Boston terrier with ventriculomegaly and early syringomyelia. (**a**) T2-weighted midsagittal brain. There is dilatation of the lateral (blue star), third (pink arrow), and fourth ventricle (blue arrow) with syringomyelia (green arrow). There is hyperdynamic flow of the CSF in the region of the obex, as evidenced by the hypointense fluid void sign (yellow arrow). The bulging caudal medullary velum can be appreciated (red arrow). There is marked reduction of the olfactory bulbs (white arrow), with ventral rotation of the brain because of brachycephaly, in addition to reduction of the nasal cavity. Consequently, reduced absorption of CSF through the olfactory lymphatics is suspected. (**b**) T2-weighted transverse brain at the level of the lateral aperture of the fourth ventricle (yellow arrows). The Boston Terrier in (**a**) is on the left and a normal brachycephalic dog on the right. The lateral apertures in the Boston Terrier cannot be appreciated. There is an absence of hyperintense CSF, and obstruction of these CSF pathways are suspected (images created by C. Rusbridge and S.P. Knowler).

CSF passes from the ventricular to the subarachnoid space via the lateral apertures of the fourth ventricle (in humans, referred to as the foramen of Luschka). Primates have an additional foramen of Megendie in the median plane between the fourth ventricle and cisterna magnum [67]. In species other than primates, the fourth ventricle and cisterna magna are separated by the caudal medullary velum [67]. The authors hypothesize that a reduced caudal cranial fossa volume in some brachycephalic breeds results in obstruction of this CSF pathway, contributing to the tendency for ventriculomegaly and syringomyelia (Figure 2).

## 9. CSF Disorders in Brachycephalic Animals

### 9.1. Ventriculomegaly and Hydrocephalus

#### 9.1.1. Characteristics

**Figure 3:**
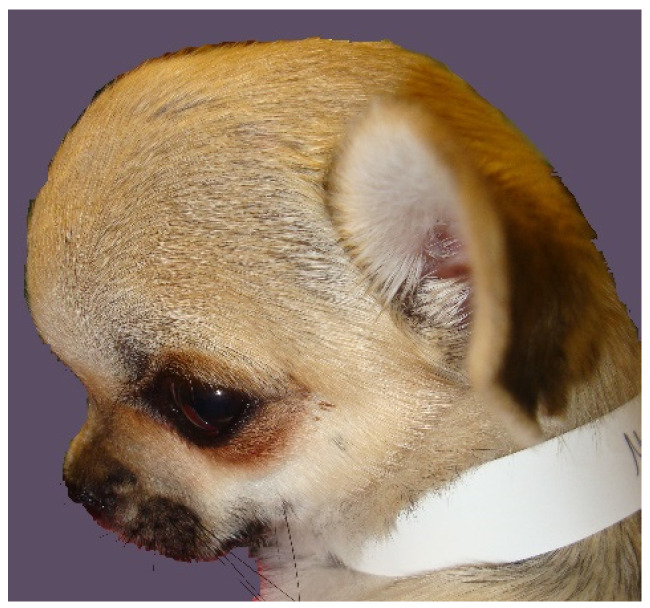
A one-year female Chihuahua presented with hydrocephalus. Clinical examination found a dome-shaped cranium with wide bregmatic fontanelle (molera). There were behavioral signs suggesting head and cranial neck pain and a hypermetric gait suggesting spinocerebellar tract or cerebellar dysfunction (photo taken by C. Rusbridge).

Hydrocephalus is defined as an active distension of the ventricular system of the brain, resulting from inadequate passage of CSF from its point of production within the cerebral ventricles to its point of absorption into the systemic circulation [68]. Ventriculomegaly describes ventricular distension in clinically normal animals. By contrast, animals with hydrocephalus are neurologically abnormal, presenting with forebrain disease and variable head or cervical pain and cerebellovestibular signs (Figure 3).

#### 9.1.2. Proposed Contributory Pathophysiology in Brachycephaly

The authors propose that the tendency for brachycephalic animals to have ventriculomegaly, and in more extreme cases hydrocephalus, is because of a combination of reduced absorption of CSF through lymphatics and altered neuroparenchymal compliance and pulsatility. Contributary factors may include reduced movement of CSF due to apnea and interruption of sleep, cranial venous stenosis, constricted CSF pathways through the lateral apertures and craniocervical junction, and intrathoracic pressure gradients. However, if there is expansion of the lateral and third ventricles only (tri-ventricular hydrocephalus) with aqueduct stenosis, then mesencephalic developmental anomalies, including fusion of the colliculi, are also likely [67].

### 9.2. Quadrigeminal Cistern Expansion

#### 9.2.1. Characteristics

**Figure 4:**
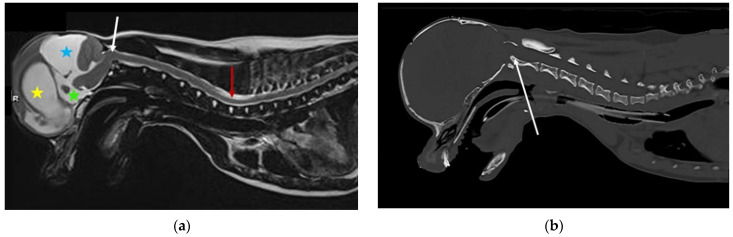
MRI and CT of a two-year-old female Chihuahua with hydrocephalus, quadrigeminal cistern expansion, and syringomyelia. (**a**) T2-weighted midsagittal brain and cervicothoracic spine. Yellow star, dilated lateral ventricle; green star, dilated third ventricle; blue star, quadrigeminal cistern expansion with compression of the cerebellum, which is herniated into the spinal canal. White arrow, craniocervical junction abnormality with cervical flexure, angulation of the odontoid peg, and compression of the craniospinal junction. Red arrow, developing cervicothoracic syrinx. (**b**) Mid-sagittal reformatted CT of the skull and cranial cervical spine. White arrow, craniocervical junction abnormality with cervical flexure, angulation of the odontoid peg, and compression of the craniospinal junction (images created by C. Rusbridge and S.P. Knowler).

The quadrigeminal cistern is a midline dilatation of the subarachnoid space, which is connected to the peri-cerebellar subarachnoid space caudally and medial cerebral hemisphere subarachnoid space laterally. It is dorsal to the third ventricle and mesencephalic aqueduct, but separated from the ventricular system by a thin membrane of pia mater and ependyma [69]. Expansion of the quadrigeminal cistern may be seen in isolation or in conjunction with dorsocaudal expansion of the third ventricle. The term supracollicular fluid collection is used to describe any expansion of CSF-filled spaces rostral to the cerebellum and dorsal to the colliculi and quadrigeminal plate (tectal plate; tectum). Supracollicular fluid collection is a common incidental finding in brachycephalic animals, but may cause clinical signs when sufficiently large enough to compress the adjacent occipital lobe or cerebellum [70] (Figure 4).

#### 9.2.2. Proposed Contributory Pathophysiology in Brachycephaly

Pathogenesis of quadrigeminal cistern expansion is unproven. Although this subarachnoid cistern is anatomically separated from the ventricular system, there is evidence that the quadrigeminal cistern is connected to the third ventricle via the velum interpositum and to the fourth ventricle via the dorsal medullary velum [71]. These valae are subarachnoid extensions, and are hypothesized to function as an alternative CSF route in circumstances of abnormal CSF circulation. In a chronic obstructive hydrocephalus rodent model (kaolin injected into the cisterna magna, causing obstruction of the lateral apertures), CSF flows from the third ventricle into the quadrigeminal cistern, and from the lateral ventricle into the ambient cistern, suggesting that communication may develop in some pathological states [72]. The authors hypothesize that, for certain brain and CSF pathway conformations, a pressure differential is created to allow one-way valve “balloon” expansion of the quadrigeminal cistern.

### 9.3. Chiari-Like Malformation Associated Pain

#### 9.3.1. Characteristics

Chiari-like malformation associated pain in dogs describes a syndrome of pain associated with brachycephaly and hindbrain herniation. It is often compared to Chiari type I and 0 malformation in humans. However, it is more like the hindbrain herniation seen with syndromic and complex craniosynostosis in humans, for example, Crouzon’s and Pfeiffer syndrome [73]. Craniofacial insufficiency, combined with occipital bone insufficiency and altered craniocervical junction conformation, results in neuroparenchymal disproportion and obstruction of the CSF channels. The most common sign is postural pain (pain being lifted or on rising and jumping), together with avoidance of exertion, signs of head and spinal pain, variable sleep disturbance, and behavioral changes [74].

#### 9.3.2. Proposed Contributory Pathophysiology in Brachycephaly

**Figure 5:**
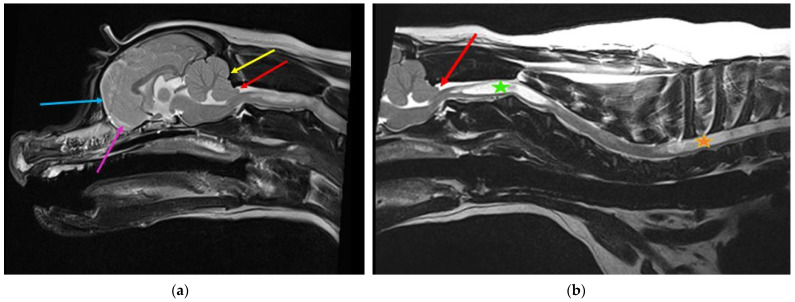
Two-year-old female Cavalier King Charles spaniel with Chiari-like malformation and syringomyelia. (**a**) T2-weighted mid-sagittal brain MRI. There is rostrotentorial crowding, giving the rostral forebrain a flattened appearance (blue arrow), with reduced size and ventrally displaced olfactory bulbs (pink arrow). The caudal fossa is reduced rostrally by the displaced forebrain and caudally by a short vertical supraoccipital bone (yellow arrow). The cerebellum is flattened against the supraoccipital bone, resulting in caudal vermal indentation and herniation into or through the foramen magnum (red arrow). (**b**) T2-weighted mid-sagittal cervicothoracic spinal MRI showing cerebellar vermis herniation (red arrow). There is wide syringomyelia in the cervical (green star) and thoracic (orange star) spinal cord. This dog was presented with signs of pain and fictive scratching (images created by C. Rusbridge and S.P. Knowler).

Many small breed dogs are predisposed to Chiari-like malformation, and may have magnetic resonance imaging (MRI) evidence of brachycephaly with hindbrain herniation. However, dogs with signs of pain have more extreme brachycephaly with craniofacial and occipital bony tissue reduction [34,75]. The reduction in the rostral cranial fossa results in rostrotentorial crowding, giving the rostral forebrain a flattened appearance with reduced ventrally displaced olfactory bulbs. The caudal fossa is reduced rostrally by the displaced forebrain and caudally by a short vertical supraoccipital bone. Some dogs have comparatively big brains [76,77]. The cerebellum is flattened against the supraoccipital bone, resulting in vermal indentation and herniation into or through the foramen magnum (Figure 5). The mechanism of the pain is controversial. The most logical mechanism is by obstruction of CSF pathways and reduced intracranial compliance, however, pain may be maintained by a maladapted trigeminocervical complex [78,79].

### 9.4. Syringomyelia

#### 9.4.1. Characteristics

Syringomyelia describes spinal cord cavitation (syrinx) with fluid similar to CSF [80]. Syringomyelia is associated with obstruction of CSF pathways, and has been reported in a variety of disorders ranging from intracranial masses to spinal arachnoid diverticulum. However, in veterinary medicine, by far the most common cause is associated with Chiari-like malformation. A small syrinx may not be associated with clinical signs. Large syringomyelia may cause signs of pain, scoliosis, weakness, and ataxia [74]. In dogs, a large syrinx in the mid cervical region that extends to the superficial dorsal horn may have signs of fictive scratching (Figure 5) [81].

#### 9.4.2. Proposed Contributory Pathophysiology in Brachycephaly

In comparison to dogs with Chiari-like malformation only, dogs with syringomyelia have more extreme brachycephaly with craniocervical junction deformation, including cervical flexure, change in angulation of the odontoid peg, increased proximity of the atlas to the skull (often referred to as atlanto-occipital overlapping), kinking or elevation of the craniospinal junction, and loss of the cisterna magna [13,75,82,83,84]. Changes in conformation of the spinal canal and cord may also contribute [85,86]. The authors propose that syringomyelia develops due to a combination of reduced CSF absorption though nasal lymphatics, reduced venous drainage, altered neuroparenchymal compliance, and reduced CSF movement through the lateral apertures or craniocervical junction. Curvature of the spinal canal and intrathoracic pressure gradient may contribute especially in the thoracic spinal cord. The mechanism of development of syringomyelia is controversial. The most accepted theory is that subarachnoid space obstruction results in a mismatch in timing between the arterial pulse peak pressure and CSF pulse peak pressure. The perivascular space changes in size during the cardiac cycle, and is widest when the spinal arteriole pressure is low. Earlier arrival of peak CSF pressure compared to peak spinal arterial pressure encourages the flow of CSF into the perivascular space, which acts as a leaky one-way valve. From the perivascular space, fluid flows into the central canal, ultimately resulting in a syrinx [87].

## 10. Conclusions

Dogs and cats with neonatal characteristics of a reduced muzzle and brachycephaly have impaired CSF circulation, which predisposes ventriculomegaly, hydrocephalus, quadrigeminal cistern expansion, Chiari-like malformation associated pain, and syringomyelia. Reduction of the lymphatic absorption of CSF through the nasal and skull base lymphatics, in conjunction with restriction of CSF movement through the lateral apertures and craniocervical junction, are hypothesized to be key features. Cranial venous stenosis, spinal canal conformation, chronic intermittent hypoxia, and thoracic cavity pressure gradients may contribute. The ethics of choosing to breed animals with this predisposition is questionable, despite their popularity as companion animals. Education of the pet-buying public, avoiding breeding extreme animals, and screening at-risk breeding stock is recommended.

## Data Availability

No new data were created or analyzed in this study. Data sharing is not applicable to this article.

## References

[1] Packer R.M.A., O’Neill D.G., Fletcher F., Farnworth M.J. (2019). Great expectations, inconvenient truths, and the paradoxes of the dog-owner relationship for owners of brachycephalic dogs. PLoS ONE.

[2] Skipper A. (2019). The ‘Dog Doctors’ of Edwardian London: Elite Canine Veterinary Care in the Early Twentieth Century. Soc. Hist. Med..

[3] Stockyard C.R. (1941). The Genetic and Endocrinic Basis for Differences in Form and Behaviour. Anatomical Memoirs.

[4] Geiger M., Haussman S. (2016). Cranial Suture Closure in Domestic Dog Breeds and Its Relationships to Skull Morphology. Anat. Rec..

[5] Schmidt M., Volk H., Klingler M., Failing K., Kramer M., Ondreka N. (2013). Closure of the cranial base synchondroses in mesaticephalic and brachycephalic dogs in comparison to Cavalier King Charles Spaniels. Vet. Radiol. Ultrasound.

[6] Schmidt M.J., Kampschulte M., Enderlein S., Gorgas D., Lang J., Ludewig E., Fischer A., Meyer-Lindenberg A., Schaubmar A.R., Failing K. (2017). The Relationship between Brachycephalic Head Features in Modern Persian Cats and Dysmorphologies of the Skull and Internal Hydrocephalus. J. Vet. Intern. Med. Am. Coll. Vet. Intern. Med..

[7] Lindsay W.L. (1871). Insanity in the Lower Animals. Br. Foreign Med. Chir. Rev..

[8] Taylor R. (1830). Catalogue of the Hunterian Collection in the Museum of the Royal College of Surgeons in London. Part II The Pathological Preparations in a Dried State.

[9] Di Ieva A., Bruner E., Davidson J., Pisano P., Haider T., Stone S.S., Cusimano M.D., Tschabitscher M., Grizzi F. (2013). Cranial sutures: A multidisciplinary review. Child. Nerv. Syst. Chns Off. J. Int. Soc. Pediatric Neurosurg..

[10] Tubbs R.S., Bosmia A.N., Cohen-Gadol A.A. (2012). The human calvaria: A review of embryology, anatomy, pathology, and molecular development. Child. Nerv. Syst..

[11] Virchow R. (1851). Uber den cretinismus, namentilich in franken, und uber pathologische schadelformen. Verh Phys. Med. Ges. Wurzbg..

[12] Roberts T., McGreevy P., Valenzuela M. (2010). Human induced rotation and reorganization of the brain of domestic dogs. PLoS ONE.

[13] Knowler S.P., Cross C., Griffiths S., McFadyen A.K., Jovanovik J., Tauro A., Kibar Z., Driver C.J., Ragione R.M.L., Rusbridge C. (2017). Use of morphometric mapping to characterise symptomatic chiari-like malformation, secondary syringomyelia and associated brachycephaly in the cavalier king charles spaniel. PLoS ONE.

[14] Tomycz L.D., Hale A.T., George T.M. (2017). Emerging Insights and New Perspectives on the Nature of Hydrocephalus. Pediatric Neurosurg..

[15] Sato O., Bering E.A., Yagi M., Tsugane R., Hara M., Amano Y., Asai T. (1975). Bulk flow in the cerebrospinal fluid system of the dog. Acta Neurol. Scand..

[16] Orešković D., Klarica M. (2010). The formation of cerebrospinal fluid: Nearly a hundred years of interpretations and misinterpretations. Brain Res. Rev..

[17] Oi S., Di Rocco C. (2006). Proposal of “evolution theory in cerebrospinal fluid dynamics” and minor pathway hydrocephalus in developing immature brain. Child. Nerv. Syst..

[18] Ma Q., Ineichen B.V., Detmar M., Proulx S.T. (2017). Outflow of cerebrospinal fluid is predominantly through lymphatic vessels and is reduced in aged mice. Nat. Commun..

[19] Casaca-Carreira J., Temel Y., Hescham S.A., Jahanshahi A. (2018). Transependymal Cerebrospinal Fluid Flow: Opportunity for Drug Delivery?. Mol. Neurobiol..

[20] Bulat M., Klarica M. (2011). Recent insights into a new hydrodynamics of the cerebrospinal fluid. Brain Res. Rev..

[21] Jessen N.A., Munk A.S., Lundgaard I., Nedergaard M. (2015). The Glymphatic System: A Beginner’s Guide. Neurochem. Res..

[22] Albertini R., Bianchi R. (2010). Aquaporins and glia. Curr. Neuropharmacol..

[23] Thomas J.H. (2019). Fluid dynamics of cerebrospinal fluid flow in perivascular spaces. J. R. Soc. Interface R. Soc..

[24] Iliff J.J., Wang M., Liao Y., Plogg B.A., Peng W., Gundersen G.A., Benveniste H., Vates G.E., Deane R., Goldman S.A. (2012). A Paravascular Pathway Facilitates CSF Flow Through the Brain Parenchyma and the Clearance of Interstitial Solutes, Including Amyloid β. Sci. Transl. Med..

[25] Lun M.P., Monuki E.S., Lehtinen M.K. (2015). Development and functions of the choroid plexus-cerebrospinal fluid system. Nat. Rev. Neurosci..

[26] Ma Q., Decker Y., Müller A., Ineichen B.V., Proulx S.T. (2019). Clearance of cerebrospinal fluid from the sacral spine through lymphatic vessels. J. Exp. Med..

[27] Schwalbe G. (1869). Die Arachnoidalraum, ein Lymphraum und sein Zusammenhang mit dem Perichoriordalraum. Zbl Med. Wiss Zent. Fur. Die Med. Wiss..

[28] Zakharov A., Papaiconomou C., Johnston M. (2004). Lymphatic vessels gain access to cerebrospinal fluid through unique association with olfactory nerves. Lymphat. Res. Biol..

[29] Leeds S.E., Kong A.K., Wise B.L. (1989). Alternative pathways for drainage of cerebrospinal fluid in the canine brain. Lymphology.

[30] Sokolowski W., Czubaj N., Skibniewski M., Barszcz K., Kupczynska M., Kinda W., Kielbowicz Z. (2018). Rostral cranial fossa as a site for cerebrospinal fluid drainage—volumetric studies in dog breeds of different size and morphotype. BMC Vet. Res..

[31] Wagner F., Ruf I. (2021). “*Forever young*”—Postnatal growth inhibition of the turbinal skeleton in brachycephalic dog breeds (*Canis lupus familiaris*). Anat. Rec..

[32] Mollanji R., Bozanovic-Sosic R., Zakharov A., Makarian L., Johnston M.G. (2002). Blocking cerebrospinal fluid absorption through the cribriform plate increases resting intracranial pressure. American journal of physiology. Regul. Integr. Comp. Physiol..

[33] Mollanji R., Bozanovic-Sosic R., Silver I., Li B., Kim C., Midha R., Johnston M. (2001). Intracranial pressure accommodation is impaired by blocking pathways leading to extracranial lymphatics. Am. J. Physiol. Regul. Integr. Comp. Physiol..

[34] Knowler S.P., Dumas E., Spiteri M., McFadyen A.K., Stringer F., Wells K., Rusbridge C. (2020). Facial changes related to brachycephaly in Cavalier King Charles Spaniels with Chiari-like malformation associated pain and secondary syringomyelia. J. Vet. Intern. Med..

[35] Ahn J.H., Cho H., Kim J.-H., Kim S.H., Ham J.-S., Park I., Suh S.H., Hong S.P., Song J.-H., Hong Y.-K. (2019). Meningeal lymphatic vessels at the skull base drain cerebrospinal fluid. Nature.

[36] Da Mesquita S., Fu Z., Kipnis J. (2018). The Meningeal Lymphatic System: A New Player in Neurophysiology. Neuron.

[37] Louveau A., Smirnov I., Keyes T.J., Eccles J.D., Rouhani S.J., Peske J.D., Derecki N.C., Castle D., Mandell J.W., Lee K.S. (2015). Structural and functional features of central nervous system lymphatic vessels. Nature.

[38] Aspelund A., Antila S., Proulx S.T., Karlsen T.V., Karaman S., Detmar M., Wiig H., Alitalo K. (2015). A dural lymphatic vascular system that drains brain interstitial fluid and macromolecules. J. Exp. Med..

[39] Schmidt M.J., Ondreka N., Rummel C., Volk H., Sauerbrey M., Kramer M. (2012). Volume reduction of the jugular foramina in Cavalier King Charles Spaniels with syringomyelia. BMC Vet. Res..

[40] Rusbridge C., Knowler S.P., Pieterse L., McFadyen A.K. (2009). Chiari-like malformation in the Griffon Bruxellois. J. Small Anim. Pract..

[41] Brühl K., Stoeter P., Wietek B., Schwarz M., Humpl T., Schumacher R., Spranger J. (2001). Cerebral spinal fluid flow, venous drainage and spinal cord compression in achondroplastic children: Impact of magnetic resonance findings for decompressive surgery at the cranio-cervical junction. Eur. J. Pediatr..

[42] Fenn J., Schmidt M.J., Simpson H., Driver C.J., Volk H.A. (2013). Venous sinus volume in the caudal cranial fossa in Cavalier King Charles spaniels with syringomyelia. Vet. J..

[43] McGonnell I.M., Akbareian S.E. (2019). Like a hole in the head: Development, evolutionary implications and diseases of the cranial foramina. Semin. Cell Dev. Biol..

[44] Tischfield M.A., Robson C.D., Gilette N.M., Chim S.M., Sofela F.A., DeLisle M.M., Gelber A., Barry B.J., MacKinnon S., Dagi L.R. (2017). Cerebral Vein Malformations Result from Loss of Twist1 Expression and BMP Signaling from Skull Progenitor Cells and Dura. Dev. Cell.

[45] Liu N.C., Troconis E.L., Kalmar L., Price D.J., Wright H.E., Adams V.J., Sargan D.R., Ladlow J.F. (2017). Conformational risk factors of brachycephalic obstructive airway syndrome (BOAS) in pugs, French bulldogs, and bulldogs. PLoS ONE.

[46] Packer R.M., Tivers M.S. (2015). Strategies for the management and prevention of conformation-related respiratory disorders in brachycephalic dogs. Vet. Med..

[47] Rubin J.A., Holt D.E., Reetz J.A., Clarke D.L. (2015). Signalment, clinical presentation, concurrent diseases, and diagnostic findings in 28 dogs with dynamic pharyngeal collapse (2008–2013). J. Vet. Intern. Med..

[48] Facin A.C., Uscategui R.A.R., Maronezi M.C., Pavan L., Menezes M.P., Montanhim G.L., Camacho A.A., Feliciano M.A.R., Moraes P.C. (2020). Liver and spleen elastography of dogs affected by brachycephalic obstructive airway syndrome and its correlation with clinical biomarkers. Sci. Rep..

[49] Packer R.M.A., Hendricks A., Tivers M.S., Burn C.C. (2015). Impact of Facial Conformation on Canine Health: Brachycephalic Obstructive Airway Syndrome. PLoS ONE.

[50] Yamada S., Miyazaki M., Yamashita Y., Ouyang C., Yui M., Nakahashi M., Shimizu S., Aoki I., Morohoshi Y., McComb J.G. (2013). Influence of respiration on cerebrospinal fluid movement using magnetic resonance spin labeling. Fluids Barriers CNS.

[51] Feinberg D.A., Mark A.S. (1987). Human brain motion and cerebrospinal fluid circulation demonstrated with MR velocity imaging. Radiology.

[52] Bert R.J., Settipalle N., Tiwana E., Muddasani D., Nath R., Wellman B., Mihlon F., Negahdar M., Amini A., Boakye M. (2019). The relationships among spinal CSF flows, spinal cord geometry, and vascular correlations: Evidence of intrathecal sources and sinks. American journal of physiology. Regul. Integr. Comp. Physiol..

[53] Bothwell S.W., Janigro D., Patabendige A. (2019). Cerebrospinal fluid dynamics and intracranial pressure elevation in neurological diseases. Fluids Barriers CNS.

[54] Sugita Y., Iijima S., Teshima Y., Shimizu T., Nishimura N., Tsutsumi T., Hayashi H., Kaneda H., Hishikawa Y. (1985). Marked episodic elevation of cerebrospinal fluid pressure during nocturnal sleep in patients with sleep apnea hypersomnia syndrome. Electroencephalogr. Clin. Neurophysiol..

[55] Román G.C., Jackson R.E., Fung S.H., Zhang Y.J., Verma A.K. (2019). Sleep-Disordered Breathing and Idiopathic Normal-Pressure Hydrocephalus: Recent Pathophysiological Advances. Curr. Neurol. Neurosci. Rep..

[56] Hauglund N.L., Pavan C., Nedergaard M. (2020). Cleaning the sleeping brain—The potential restorative function of the glymphatic system. Curr. Opin. Physiol..

[57] Reidenberg J.S., Laitman J.T. (1991). Effect of basicranial flexion on larynx and hyoid position in rats: An experimental study of skull and soft tissue interactions. Anat. Rec..

[58] Neelapu B.C., Kharbanda O.P., Sardana H.K., Balachandran R., Sardana V., Kapoor P., Gupta A., Vasamsetti S. (2017). Craniofacial and upper airway morphology in adult obstructive sleep apnea patients: A systematic review and meta-analysis of cephalometric studies. Sleep Med. Rev..

[59] Hoyte D.A.N., Dixon A.D., Hoyte D.A.N., Ronning O. (1997). Growth of the Cranial Base. Fundamentals of Craniofacial Growth.

[60] Dubrul E.L., Laskin D.M. (1961). Preadaptive potentialities of the mammalian skull: An experiment in growth and form. Am. J. Anat..

[61] Knowler S.P., Galea G.L., Rusbridge C. (2018). Morphogenesis of Canine Chiari Malformation and Secondary Syringomyelia: Disorders of Cerebrospinal Fluid Circulation. Front. Vet. Sci..

[62] Donnally I.C., Munakomi S., Varacallo M. (2020). Basilar Invagination. StatPearls, StatPearls Publishing Copyright © 2020.

[63] Shoja M.M., Ramdhan R., Jensen C.J., Chern J.J., Oakes W.J., Tubbs R.S. (2018). Embryology of the craniocervical junction and posterior cranial fossa, part I: Development of the upper vertebrae and skull. Clin. Anat..

[64] Flanagan M.F. (2015). The Role of the Craniocervical Junction in Craniospinal Hydrodynamics and Neurodegenerative Conditions. Neurol. Res. Int..

[65] Williams B. (1993). Pathogenesis of syringomyelia. Acta Neurochir..

[66] Rai S.K., Rai P. (2015). Volume change theory for syringomyelia: A new perspective. Asian J. Neurosurg..

[67] DeLahunta A., Glass E., Kent M. (2014). Cerebrospinal Fluid and Hydrocephalous. Veterinary Neuroanatomy and Clinical Neurology.

[68] Rekate H.L. (2008). The definition and classification of hydrocephalus: A personal recommendation to stimulate debate. Cereb. Fluid Res..

[69] Bertolini G., Ricciardi M., Caldin M. (2016). Multidetector computed tomographic and low-field magnetic resonance imaging anatomy of the quadrigeminal cistern and characterization of supracollicular fluid accumulations in dogs. Vet. Radiol. Ultrasound.

[70] Matiasek L.A., Platt S.R., Shaw S., Dennis R. (2007). Clinical and magnetic resonance imaging characteristics of quadrigeminal cysts in dogs. J. Vet. Intern. Med. Am. Coll. Vet. Intern. Med..

[71] Fenstermacher J.D., Ghersi-Egea J.F., Finnegan W., Chen J.L. (1997). The rapid flow of cerebrospinal fluid from ventricles to cisterns via subarachnoid velae in the normal rat. Acta neurochirurgica. Supplement.

[72] Yoon J.S., Nam T.K., Kwon J.T., Park S.W., Park Y.S. (2015). CSF flow pathways through the ventricle-cistern interfaces in kaolin-induced hydrocephalus rats-laboratory investigation. Child. Nerv. Syst. Chns Off. J. Int. Soc. Pediatric Neurosurg..

[73] Rusbridge C. (2020). New considerations about Chiari-like malformation, syringomyelia and their management. Practice.

[74] Rusbridge C., McFadyen A.K., Knower S.P. (2019). Behavioral and clinical signs of Chiari-like malformation-associated pain and syringomyelia in Cavalier King Charles spaniels. J. Vet. Intern. Med..

[75] Knowler S.P., Kiviranta A.-M., McFadyen A.K., Jokinen T.S., La Ragione R.M., Rusbridge C. (2017). Craniometric analysis of the hindbrain and craniocervical junction of chihuahua, affenpinscher and cavalier king charles spaniel dogs with and without syringomyelia secondary to chiari-like malformation. PLoS ONE.

[76] Cross H.R., Cappello R., Rusbridge C. (2009). Comparison of cerebral cranium volumes between cavalier King Charles spaniels with Chiari-like malformation, small breed dogs and Labradors. J. Small Anim. Pract..

[77] Shaw T.A., McGonnell I.M., Driver C.J., Rusbridge C., Volk H.A. (2012). Increase in cerebellar volume in Cavalier King Charles Spaniels with Chiari-like malformation and its role in the development of syringomyelia. PLoS ONE.

[78] Vadivelu S., Bolognese P., Milhorat T.H., Mogilner A.Y. (2011). Occipital neuromodulation for refractory headache in the Chiari malformation population. Prog. Neurol. Surg..

[79] Alperin N., Loftus J.R., Oliu C.J., Bagci A.M., Lee S.H., Ertl-Wagner B., Sekula R., Lichtor T., Green B.A. (2015). Imaging-Based Features of Headaches in Chiari Malformation Type I. Neurosurgery.

[80] Brodbelt A., Flint G., Rusbridge C. (2014). The Biochemistry of Syringomyelia. Syringomyelia: A Disorder of CSF Circulation.

[81] Nalborczyk Z.R., McFadyen A.K., Jovanovik J., Tauro A., Driver C.J., Fitzpatrick N., Knower S.P., Rusbridge C. (2017). MRI characteristics for “phantom” scratching in canine syringomyelia. BMC Vet. Res..

[82] Marino D.J., Loughin C.A., Dewey C.W., Marino L.J., Sackman J.J., Lesser M.L., Akerman M.B. (2012). Morphometric features of the craniocervical junction region in dogs with suspected Chiari-like malformation determined by combined use of magnetic resonance imaging and computed tomography. Am. J. Vet. Res..

[83] Cerda-Gonzalez S., Olby N.J., Griffith E.H. (2015). Medullary position at the craniocervical junction in mature cavalier king charles spaniels: Relationship with neurologic signs and syringomyelia. J. Vet. Intern. Med. Am. Coll. Vet. Intern. Med..

[84] Kiviranta A.-M., Rusbridge C., Laitinen-Vapaavuori O., Hielm-Björkman A., Lappalainen A.K., Knowler S.P., Jokinen T.S. (2017). Syringomyelia and Craniocervical Junction Abnormalities in Chihuahuas. J. Vet. Intern. Med..

[85] Cirovic S., Lloyd R., Jovanovik J., Volk H.A., Rusbridge C. (2018). Computer simulation of syringomyelia in dogs. BMC Vet. Res..

[86] Sparks C.R., Robertson I., Olby N.J. (2019). Morphometric analysis of spinal cord termination in Cavalier King Charles Spaniels. J. Vet. Intern. Med..

[87] Stoodley M., Flint G., Rusbridge C. (2014). The Filling Mechanism. Syringomyelia: A Disorder of CSF Circulation.

